# INH14, a Small‐Molecule Urea Derivative, Inhibits the IKKα/β‐Dependent TLR Inflammatory Response

**DOI:** 10.1002/cbic.201800647

**Published:** 2019-01-29

**Authors:** Meinrad Drexel, Johannes Kirchmair, Sandra Santos‐Sierra

**Affiliations:** ^1^ Department of Pharmacology Medical University of Innsbruck 6020 Innsbruck Austria; ^2^ Department of Chemistry University of Bergen 5020 Bergen Norway; ^3^ Computational Biology Unit (CBU) University of Bergen 5020 Bergen Norway; ^4^ Zentrum für Bioinformatik Bundesstrasse 43 20146 Hamburg Germany; ^5^ Section of Biochemical Pharmacology Medical University Innsbruck Peter Mayr Strasse 1 6020 Innsbruck Austria

**Keywords:** drug discovery, inflammation, inhibitors, proteins, receptors

## Abstract

*N*‐(4‐Ethylphenyl)‐*N′‐*phenylurea (INH14) is a fragment‐like compound that inhibits the toll‐like receptor 2 (TLR2)‐mediated inflammatory activity and other inflammatory pathways (i.e., TLR4, TNF‐R and IL‐1R). In this study, we determined the molecular target of INH14. Overexpression of proteins that are part of the TLR2 pathway in cells treated with INH14 indicated that the target lay downstream of the complex TAK1/TAB1. Immunoblot assays showed that INH14 decreased IkBα degradation in cells activated by lipopeptide (TLR2 ligand). These data indicated the kinases IKKα and/or IKKβ as the targets of INH14, which was confirmed with kinase assays (IC_50_ IKK*α*=8.97 μm; IC_50_ IKK*β*=3.59 μm). Furthermore, in vivo experiments showed that INH14 decreased TNFα formed after lipopeptide‐induced inflammation, and treatment of ovarian cancer cells with INH14 led to a reduction of NF‐kB constitutive activity and a reduction in the wound‐closing ability of these cells. These results demonstrate that INH14 decreases NF‐kB activation through the inhibition of IKKs. Optimization of INH14 could lead to potent inhibitors of IKKs that might be used as antiinflammatory drugs.

## Introduction

Toll‐like receptors (TLRs) are the most thoroughly studied innate immune receptors. They recognize pathogen‐associated molecular patterns (PAMPs), which are molecular structures conserved among different pathogens.^1^ They also recognize host‐derived molecules named alarmins (danger‐associated molecular patterns (DAMPSs)), which mediate sterile inflammation after trauma, stress, and injury.^2^ Ligation of PAMPs and DAMPs by TLRs in antigen‐presenting cells, such as macrophages or dendritic cells, leads to the activation of transcription factors (e.g., nuclear factor‐kappa B (NF‐kB) or activator protein 1 (AP‐1)).^3^ These transcription factors promote the production of cytokines and chemokines. These, in turn, can lead to the activation of the adaptive immune system.^4^ It has been suggested that the endogenous ligand‐recognition response could be the underlying mechanism of inflammatory processes observed in autoimmune diseases (e.g., systemic lupus erythematosus, psoriasis) and cancer.^5^ Therefore, it might be of advantage to use small‐molecule inhibitors to block TLR activity prior to the manifestation of chronic inflammation.^6^ In this regard, previous studies have demonstrated that the selection of small‐molecules^7^ and peptides^8^ that inhibit the inflammatory response mediated by several TLRs might be successfully accomplished.

The transcription factor NF‐kB is involved in the expression of proinflammatory genes (e.g., cytokines and chemokines), and therefore, is a master regulator of inflammation. NF‐kB is activated in response to various stimuli, such as infections and stress signals.^9^ Its dysregulation is associated with a variety of diseases, such as atherosclerosis, infections, and cancer progression.^10^ Activating signals for TLR or TNF‐R receptors lead to phosphorylation in specific serine residues in the activation loop of the IkB (inhibitor of NF‐kB)‐kinases IKKα and IKKβ.^11^ In turn, the active IKK complex phosphorylates IkBα at Ser32 and Ser36, which leads to its degradation by ubiquitin‐activating enzymes and to the liberation of NF‐kB.^12^ The cytoplasmic NF‐kB then translocates into the nucleus to initiate the expression of over 500 genes involved in inflammation, carcinogenesis, and apoptosis.^13^ Different triggers lead to either the activation of the NF‐kB canonical pathway (NF‐kB1 (p50/105)), which is mediated by IKKα/β/γ, or the noncanonical pathway mediated by IKKα, which leads to activation of NF‐kB2 (p52/p100).^14^ A substantial number of small‐molecule inhibitors of IKKα and IKKβ have been reported, to date. These can be classified as adenosine triphosphate (ATP) analogues, allosteric effectors, and compounds that interact with the Cys179 residue in the activation loop of IKKβ.^13^ However, only a few of these inhibitors are selective for either of these two kinases. The design of selective small‐molecule inhibitors of IKKα and IKKβ has proven to be challenging because the active sites of both enzymes share high structural homology.^15^

**Figure 1:**
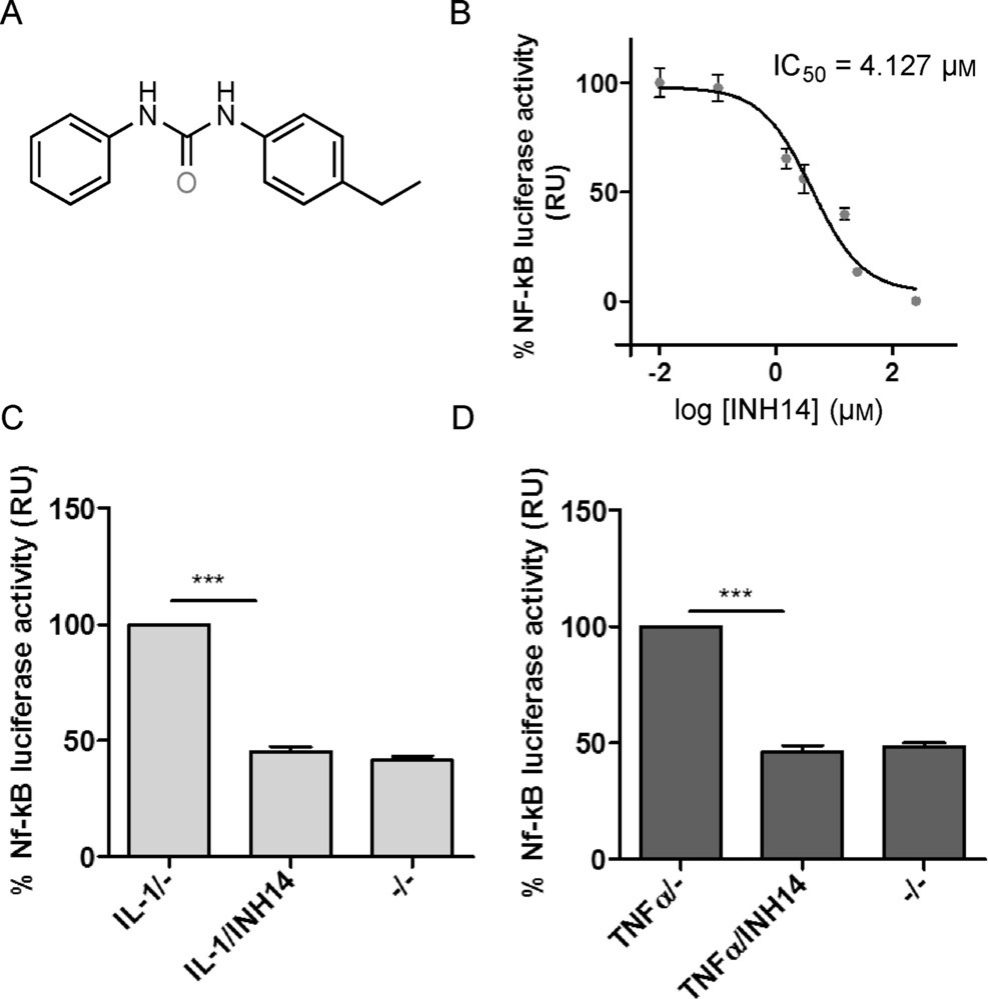
A) Chemical structure of INH14. B) HEK293‐TLR2 cells transfected with the NF‐kB reporter plasmid *Elam.luc* (15 ng per well) and a constitutive *Renilla* expression plasmid (15 ng per well). The cells were incubated with increasing concentrations of INH14 or vehicle, and stimulated with the TLR2 ligand P3 (200 ng mL^−1^). After 5 h, the level of NF‐kB activity was quantified by measuring the chemiluminescence produced by firefly luciferase in the cell lysates. The luciferase activity was normalized to the *Renilla* activity and expressed as a percentage of P3 stimulation. The points represent the mean and standard error of the mean (SEM) of three independent experiments in triplicate. The IC_50_ value was obtained by fitting of the sigmoidal dose–response plot. HEK293 cells were transfected as in B) and treated with INH14 (10 μm) or vehicle, and subsequently stimulated with C) IL‐1β (25 ng mL^−1^) or D) TNFα (50 ng mL^−1^). Afterward, the chemiluminescence signal produced by luciferase activation was measured and normalized to the *Renilla* values. The bars represent the mean and SEM of three independent experiments (statistical significance calculated by the unpaired Student t‐test; *** *p*< 0.001). RU: relative units. (–/–): incubation with the vehicle used to dissolve INH14 (DMSO) and incubation with the vehicle used to dissolve IL‐1 and TNFα (serum‐free media).

In previous studies, we reported a collection of small molecules (1–8; subsequently named AT1–AT8 for antagonist) that antagonized TLR2 activity in human cells: HEK293 cells overexpressing TLR2 (HEK293‐TLR2) and primary monocytes.7a As part of these screening efforts, INH14 (inhibitor14: *N*‐(4‐ethylphenyl)‐*N′‐*phenylurea; Figure 1 A) was identified as an inhibitor of TLR2‐mediated NF‐kB activation. However, preliminary data indicated that the inhibitory activity of the compound on TLR2 was not linked to direct interaction with that protein, as was the case for AT1–AT8, for which reason it was not reported in our previous work.

The purpose of the current study was to unravel the mechanism of inhibition of INH14 downstream of TLR2. To achieve this aim, we utilized transcriptional assays to identify potential target proteins and employed target‐based assays for confirmation. We also derived the likely binding mode of INH14 for the kinase IKKβ. In addition, we conducted in vivo experiments to evaluate the anti‐inflammatory effect of INH14. Importantly, INH14 decreased the NF‐kB constitutive activity in ovarian cancer cells. Overall, this makes INH14 a promising starting point for the development of potent and selective inhibitors of these central kinases.

## Results

### Inhibition of TLR2‐dependent NF‐kB activation by INH14

INH14 (Figure 1 A) is a fragment‐like compound with a molecular weight of only 240 Da. It mainly consists of a biaryl urea scaffold that is common to a number of kinase inhibitors; in particular, compounds addressing the epidermal growth factor receptor (EGFR)^16^ or the vascular endothelial growth factor receptor 2 (VEGFR‐2).16a, 16b, 17

To confirm that the inhibitory activity of INH14 on TLR2 signaling was dose dependent, we transfected HEK293‐TLR2 cells with a luciferase reporter tandem: the NF‐kB‐dependent reporter plasmid *Elam.luc* and the constitutively active *Renilla* plasmid (to normalize for transfection efficiency). After incubation of the cells with increasing concentrations of INH14, these were stimulated with the TLR2 ligand triacylated lipopeptide Pam3CSK4 (P3). Chemiluminescence measurements indicated that INH14 reduced TLR2‐mediated NF‐kB activity in a dosedependent manner, with a half‐inhibitory concentration of 4.127 μm (Figure 1 B). We obtained parallel results if the cells were stimulated with diacylated lipopeptide Pam2CSK4 (P2; not shown).

### Inhibition of TNFα and IL‐1 signaling by INH14

To assess the selectivity of INH14 for TLR2 inhibition, we tested the effect of INH14 in the activity of two receptors related to TLR signaling. IL‐1R shares a similar signaling pathway (MyD88‐dependent) and TNF‐R, although working through different upstream signaling components, converges at the level of the complex formed by TAK1/TAB1 and downstream proteins.^18^ HEK293 cells were transfected with *Elam.luc* and *Renilla* plasmids, as described above. The cells were treated for 1 h with INH14 and then stimulated with TNFα or IL‐1β. Unexpectedly, INH14 inhibited NF‐kB activation obtained in both cases (Figure 1 C, D). Thus, we hypothesized that INH14 might be a cell‐permeable small molecule that could interfere with the signaling downstream of TLR2, IL‐1R, and TNF‐R.

### Inhibition of TNFα production by INH14 following TLR2 or TLR4 stimulation

**Figure 2:**
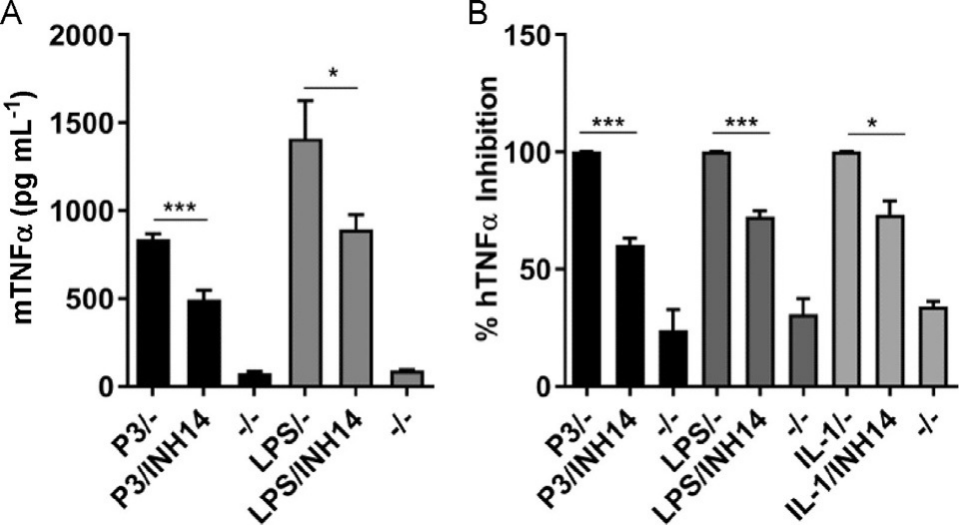
A) RAW264.7 mouse macrophages were preincubated with INH14 (15 μm) or vehicle and, after 1 h, stimulated with P3 (200 ng mL^−1^), lipopolysaccharide (LPS; 100 ng mL^−1^), or vehicle for 9 h. Then TNFα secreted into the supernatant was quantified by means of ELISA. The bars represent the mean and SEM of three independent experiments. B) Human monocytes were isolated and pretreated with INH14 (15 μm) or vehicle. After 1 h, the cells were stimulated with P3 (200 ng mL^−1^), LPS (100 ng mL^−1^), IL‐1β (50 ng mL^−1^), or vehicle and TNFα secreted into the supernatant after overnight incubation was quantified as above. The bar graphs represent the mean and SEM of the values obtained from six different donors for P3, four for LPS, and three for IL‐1β, (*** *p*<0.001; * *p*<0.05 by unpaired t‐test). (–/–): incubation with the vehicle used to dissolve INH14 (DMSO) and incubation with the vehicle used to dissolve P3 and LPS (serum‐free media).

To further investigate whether INH14 decreased the TLR2‐mediated proinflammatory activity, we tested the capacity of the compound to inhibit human and mouse TNFα production after TLR2 stimulation. Human primary monocytes or mouse RAW264.7 macrophages were incubated with INH14, and then stimulated with P3. The amount of TNFα secreted in the supernatant was quantified by means of ELISA. As shown in Figure 2 A, INH14 reduced TNFα production by mouse macrophages after P3 stimulation from (837±30.28) to (496.6±50.69) pg mL^−1^. The inhibitory effect in TLR2 was more pronounced than that obtained after stimulation of TLR4 with LPS (from (1411±214.3) to (892.8±84.71) pg mL^−1^). The reduction in TNFα production by other MyD88‐dependent TLRs after treatment with INH14 has also been confirmed (i.e., TLR7/8, TLR5; Figure S1 in the Supporting Information). Additionally, we found a decrease in the production of TNFα by human monocytes after INH14 treatment if they were stimulated with P3 (reduced to 60.43 %), LPS (72.62 %), or IL‐1 (73.30 %; Figure 2 B).

### Locating the target of INH14 downstream of TLR2

**Figure 3:**
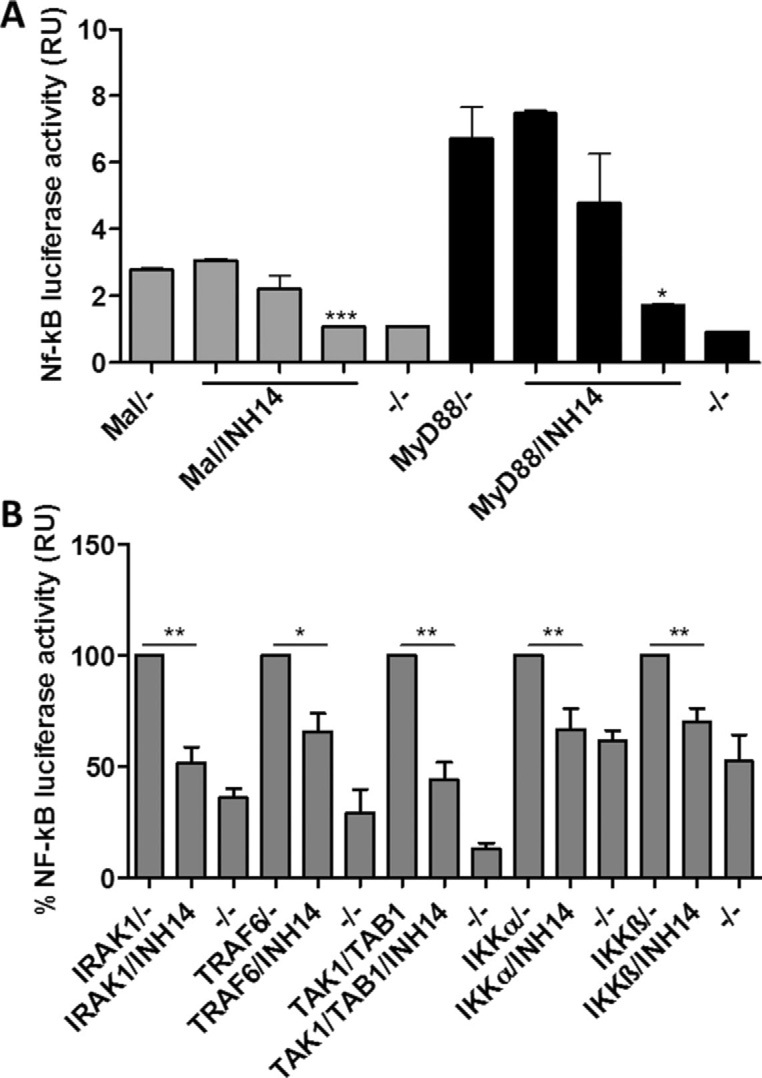
A) HEK293 cells transfected with an NF‐kB reporter plasmid and plasmids encoding the adaptor proteins Mal (5 ng per well) or MyD88 (5 ng per well) were incubated with INH14 (1, 10, 25 μm). Then, the luciferase activity was recorded, and the luciferase values were normalized to the *Renilla* values. B) HEK293 cells were treated as in A), but transfected with plasmids encoding IRAK1 (20 ng per well), TRAF6 (80 ng per well), TAK1/TAB1 (60 ng per well each), IKKα (100 ng per well), or IKKβ (100 ng per well). Then they were incubated with INH14 (15 μm), and the luciferase activity was measured as indicated in A). RU: relative units. The bars represent mean and SEM of three independent experiments. (*** *p*<0.001; ** *p*<0.01; * *p*<0.05 by unpaired t‐test). (−) incubation with the vehicle used to dissolve INH14 (DMSO). (–/–): Transfection with mock plasmid and incubation with the vehicle used to dissolve INH14 (DMSO).

Our primary hypothesis was that INH14 was an antagonist of TLR2. However, our results indicated that INH14 inhibited not only TLR2 activity, but also TLR2‐related (TLR4 and IL‐1R) and unrelated pathways (TNF‐R). Although we have not determined the permeability coefficient of INH14, our results indicate that it might be a cell‐permeable molecule. Thus, we explored at which level of the mentioned pathways the compound was effective. Overexpression of proteins described to be downstream of TLRs leads to NF‐kB activation independently of TLR stimulation.^19^ Hence, we transiently transfected HEK293 cells with plasmids encoding the TLR adaptor TIRAP/Mal, *Elam.luc*, and *Renilla*. Then, we incubated the cells with INH14 for 5 h and measured the luciferase activity. As shown in Figure 3 A, INH14 decreased the NF‐kB activity attained with Mal expression in a dose‐dependent manner. Likewise, we transfected HEK293 cells with a plasmid encoding MyD88, the next adaptor downstream of TLR2. Figure 3 A shows that INH14 also decreased the NF‐kB activity induced by MyD88.

### INH14 inhibition downstream of TAK1/TAB1

The signaling downstream of TLR2 converges at the level of IRAK/TRAF6/TAK1‐TAB1 with other TLR pathways (e.g., TLR5, MyD88‐dependent branch of TLR4 signaling). Following TAK1‐TAB1 activation, several kinases might be activated: 1) mitogen‐activated protein kinases (MAPKs), which lead to the phosphorylation of JNK, p38, and ERK and activation of the transcription factor AP‐1; and 2) IKKα/β/γ, which leads to IkBα degradation and NF‐kB translocation into the nucleus.^20^

We transfected HEK293 cells with IRAK1‐, TRAF6‐, TAK1/TAB1‐, IKKα‐, or IKKβ‐expressing plasmids and *Elam.luc* and *Renilla* (Figure 3 B). The cells were then treated with INH14 to assess its effect on the NF‐kB transcriptional activity induced by overexpression of the mentioned proteins. In all cases, INH14 reduced the activation of NF‐kB.

**Figure 4:**
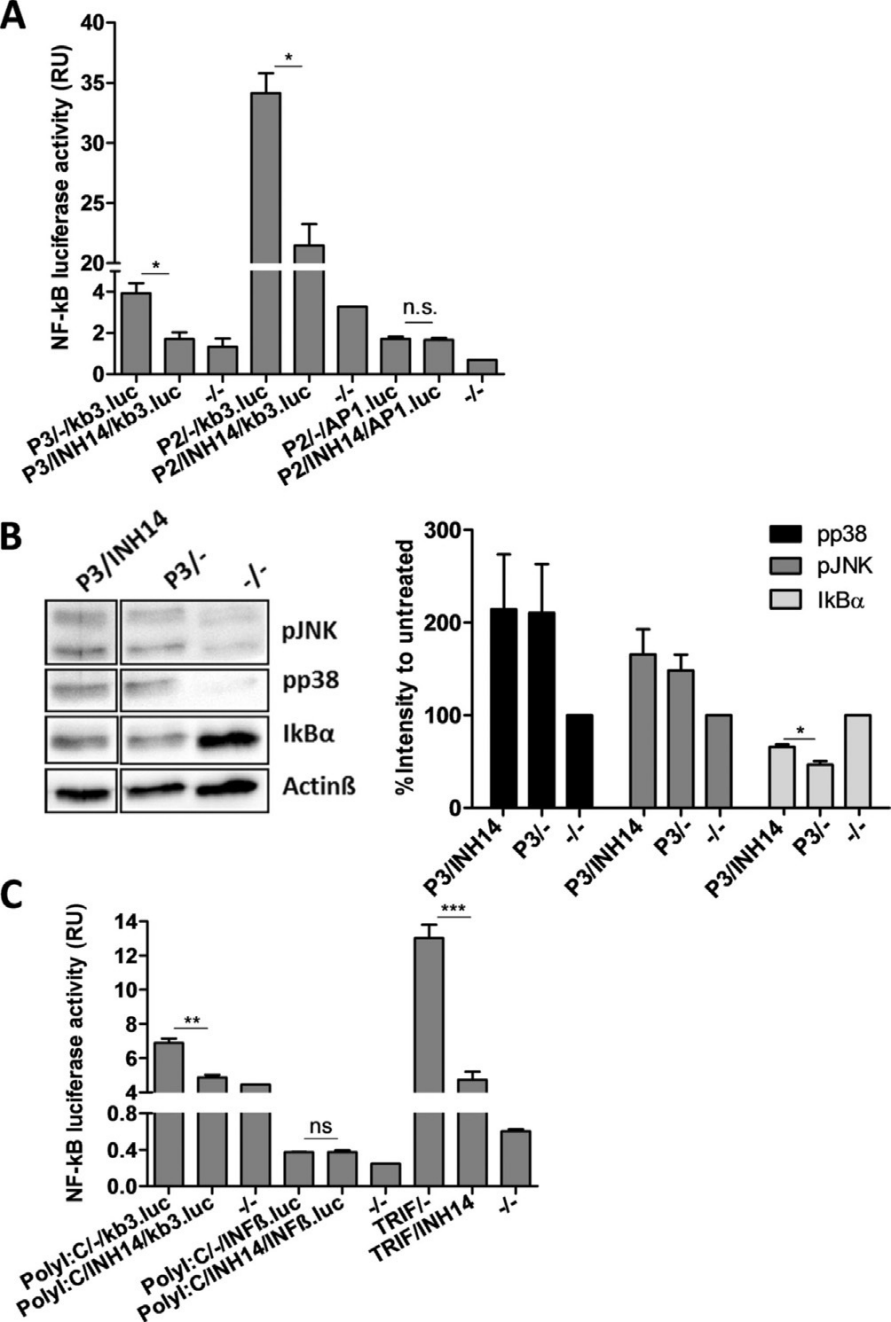
A) HEK293‐TLR2 cells were transfected with *kb3.luc* or *AP1.luc* and *Renilla* plasmids, and they were treated for 1 h with INH14 (15 μm) and stimulated with P3 (200 ng mL^−1^) or P2 (50 ng mL^−1^). After 5 h, the chemiluminescence produced by luciferase was measured and normalized to the chemiluminescence formed by *Renilla*. Bars represent the mean and SEM of three independent experiments. B) HEK293‐TLR2 cells were incubated with INH14 (25 μm) or vehicle, and then they were stimulated with P3 (200 ng mL^−1^). The effect of INH14 on the phosphorylation of JNK, p38, or IkBα degradation was assessed by means of immunoblotting with the corresponding antibodies. Different lanes were run in the same gel, but distant from each other (full immunoblot in Figure S2). The band intensity was quantified and normalized to Actinβ intensity. Three independent immunoblots were quantified (the bottom panel represents the mean±SEM); * *p*<0.05 by unpaired t‐test. C) HEK293 cells transfected with *kb3.luc*, *INF‐β.luc*, or TRIF (20 ng per well) and *kb3.luc* were incubated with INH14 (15 μm), and then they were stimulated with PolyI:C (10 μg mL^−1^) in the first two cases or vehicle (for TRIF). The luciferase activity was assessed as in A). Bars represent mean of three independent experiments in duplicate and SEM. (*** *p*<0.001; ** *p*<0.01; * *p*<0.05 by unpaired t‐test). (–/–): incubation with the vehicle used to dissolve INH14 (DMSO) and incubation with the vehicle used to dissolve P2 and P3 (serum‐free media).

The E‐selectin promotor in the *Elam.luc* reporter plasmid consists of three NF‐kB and two AP‐1 binding sites.^21^ Therefore, we next analyzed the effect of INH14 in independent activation of both transcription factors. HEK293‐TLR2 cells were transfected with the reporter plasmid *kb3.luc* (which contains three NF‐kB binding sites in the luciferase promoter), and then they were stimulated with P2 or P3 (Figure 4 A). In both cases, the NF‐kB activity obtained after TLR2 stimulation was reduced if the cells were treated with INH14. Next, we transfected HEK293‐TLR2 with an AP1‐dependent luciferase reporter plasmid (*AP1.luc*). Incubation of the cells with INH14 before treatment with P2 did not reduce the AP‐1 transcriptional activity (Figure 4 A). Due to the observed inhibition of IKKα/β activation of NF‐kB by INH14 (Figure 3 B), and the inhibition of NF‐kB activity (but not AP‐1) following TLR2 activation, we postulated that INH14 exerted its inhibitory effect at the level of IKKα/β.

To confirm our hypothesis, we monitored the activation of proteins downstream of MAPKKs or IKKα/β. To do so, HEK293‐TLR2 cells were treated with INH14 and then stimulated with P3. Immunoblotting analysis showed that the phosphorylation of p38 and JNK was not affected by INH14 treatment, whereas IkBα degradation was significantly reduced (Figure 4 B). This result further indicated IKKα/β as the possible target of INH14.

We next proceeded to discard an effect of INH14 in other proteins involved in TLR signaling different from that of the MyD88‐dependent pathway. Thus, we tested the effect of INH14 in the endosomal receptor TLR3 pathway. TLR3 stimulation by polyI:C leads to the activation of the three transcription factors NF‐kB, AP‐1, and IRF3. We used HEK293 cells to test the activation of TLR3 because they expressed this receptor, even though at low levels.^22^ HEK293 cells were transfected with reporter plasmids that encoded promoters with binding sites for the above‐mentioned transcription factors (i.e., *kb3.luc*, *INFβ.luc*, and *AP1.luc*). Then we assessed the effect of INH14 on luciferase activity after stimulation with PolyI:C (Figure 4 C). INH14 decreased the transcriptional activity of NF‐kB, but not of IRF3. Nevertheless, we did not obtain a detectable signal with the PolyI:C stimulation of cells transfected with the *AP‐1.luc* plasmid (not shown). Previous studies have shown that, after TLR3 ligation, the signaling axis TRIF‐RIP1‐TAK1/TAB1 leads to IKKα/β activation.^23^ Thus, we transfected a plasmid encoding the TRIF adaptor in HEK293 cells, which produced a similar result after incubation with INH14 (Figure 4 C). These data indicate a downstream target of INH14 in the TLR3 pathway also present in the previously identified pathways (i.e., TLR2, TLR4, IL‐1R, TNF‐R), most likely IKKα/β.

### IKKα and IKKβ as targets of INH14

**Figure 5:**
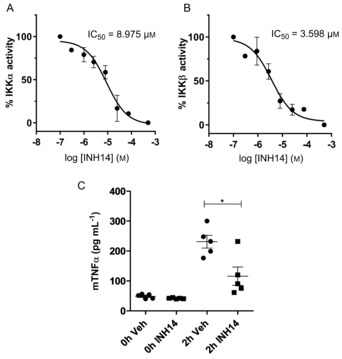
A) IKKα (15 ng per reaction) or B) IKKβ (20 ng per reaction) were incubated with ATP (50 or 25 μm, respectively) and substrate peptide (0.2 ng mL^−1^) in the presence of vehicle or increasing concentrations of INH14 at room temperature for 1 h. The graphs represent the mean and SEM of two independent experiments in duplicates. The IC_50_ value was obtained by fitting of the sigmoidal dose–response curve. C) Mice were intraperitoneally pretreated with INH14 (5 μg g^−1^) or vehicle (DMSO/NaCl) for 1 h. Then they were i.p. injected with P2 (2.5 μg g^−1^). Vein blood was taken at time 0 and 2 h after P2 injection. The TNFα level was quantified by means of ELISA. The statistical significance was assessed with the unpaired Student t‐test. * *p*<0.05.

Our previous experiments in cellulo indicated a high degree of certainty that IKKs were the cellular targets of INH14. Both kinases have been described to be involved in different cellular functions: IKKα is essential in the noncanonical NF‐kB pathway, the deregulation of which is associated with lymphoid malignancies, and IKKβ is active in the NF‐kB canonical pathway in control of immune responses^24^ and additional functions such as angiogenesis or insulin resistance.^25^ To investigate the kinase‐inhibitory activity of INH14, we performed kinase assays with recombinant IKKα. Preincubation of the enzyme with increasing concentrations of INH14 led to a reduction of phosphorylated product (IC_50_=8.97 μm; Figure 5 A). Because several of the described IKKα inhibitors also inhibited IKKβ, we also performed kinase assays with this enzyme. As seen in Figure 5 B, INH14 inhibited IKKβ catalytic activity in a dose‐dependent way, with an even lower IC_50_ than that obtained for IKKα (IC_50_=3.59 μm). Taken together, these results indicate that INH14 is a potent inhibitor of IKKα/β, and therefore, an inhibitor of the canonical and noncanonical NF‐kB pathways. However, unrelated kinases, which have not been tested in this study, might also be targeted, even if with lower affinity.

### Reduced lipopeptide‐induced inflammation in mice by INH14

Intraperitoneal lipopeptide injection in mice leads to a maximum TNFα production 2 h after injection.^26^ To study the effect of INH14 on systemic inflammation, C57BL/6J mice were intraperitoneally injected with INH14 (5 μg g^−1^), followed by i.p. injection of P2 (2.5 μg g^−1^). The TNFα in the serum of the mice was quantified by means of ELISA (Figure 5 C). Mice treated with INH14 had a decreased level of TNFα, in comparison with the control group (from (231.1±21.3) to (115.8±30.61) pg mL^−1^). Thus, the inhibition of IKKα/β by INH14 in vivo leads to a decrease in TNFα production after lipopeptide injection. During these experiments, we did not observe pathological effects in animals treated with the compound (e.g., weight loss, abnormal movements, dyspnoea).

INH14 did not affect the activation of the INF‐β promoter after TLR3 activation (Figure 4 C). Therefore, gene transcription induced by TLR3 activation through IRF3 was unaltered. Thus, although INH14 has in vivo anti‐inflammatory activity (e.g., potentially in Gram‐positive bacterial sepsis with increased TLR2 functioning), antiviral activity might not be compromised by INH14 treatment. Nevertheless, IKK inhibitors have to be carefully characterized in vivo because systemic kinase inhibition might potentially lead to septic shock.^27^ Further in vivo studies will follow to assess the effect of INH14 in another kind of inflammation model (e.g., LPS‐induced shock, inflamed paw model).

### INH14 is not toxic to primary human monocytes, but inhibits the growth of ovarian cancer cells

**Figure 6:**
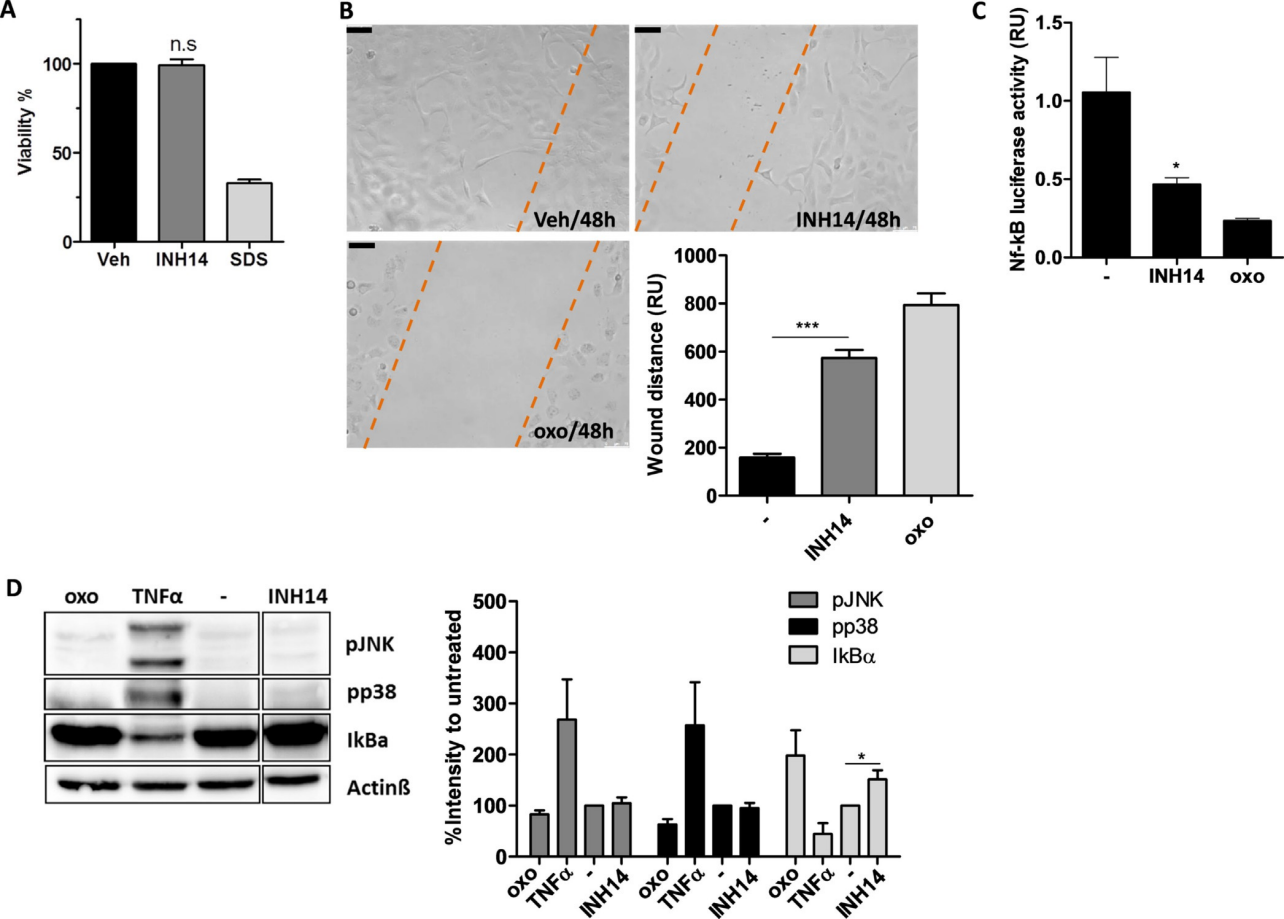
A) Human monocytes from four independent donors were incubated overnight with INH14 (20 μm), vehicle (Veh; DMSO), or SDS (0.02 %). Then, the cell viability was assessed through the CCK‐8 assay. n.s: nonsignificant difference between INH14 treatment and vehicle. B) Bright‐field photographs of the wound‐healing assay with SKOV3 cells treated with INH14 (20 μm), vehicle ((−); DMSO), or 5*Z*‐7‐oxozeaenol (oxo; 20 μm) for 48 h. Scale bars: 75 μm. The bar graph represents the quantification of four independent experiments (mean and SEM). *** *p*<0.001 by the Student t‐test. C) SKOV3 cells were transfected with the NF‐KB reporter *Elam.luc* and *Renilla* plasmids, and the effect of INH14 (20 μm) in constitutive NF‐kB activation was measured through chemiluminescence detection. D) SKOV3 cells were incubated with INH14 (25 μm), 5*Z*‐7‐oxozeaenol (25 μm), TNFα (0.1 μg mL^−1^), or vehicle (−). The phosphorylation of JNK and p38, and the degradation of IkBα were assessed with anti‐phospho JNK, anti‐phospho p38, and anti‐IkBα antibodies and normalized to the Actinβ intensity values. In the right panel of D), the bar graph represents the mean and SEM of three independent experiments. The INH14‐treated sample was run in the same gel, but in a separate lane (full immunoblot in Figure S2).

We next investigated if INH14 was toxic for human primary immune cells. Monocytes from healthy volunteers were prepared and seeded in 96‐well plates. Then, the cells were treated overnight with INH14 or with vehicle. The next day, cell viability was assessed through the CCK‐8 assay (dehydrogenase activity in viable cells), which showed that INH14 (20 μm) was not toxic to the cells, in comparison to vehicle incubation (Figure 6 A).

The important role of IKKs in cancer regulation is backed up by multiple studies.^28^ IKKs regulate NF‐kB activation, which, in turn, controls crucial steps in tumor development, such as transformation, survival, proliferation, and metastasis. Moreover, silencing different IKK subunits or their pharmacological inhibition promote cell death and sensitize cancer cells to chemotherapeutic agents.^29^ Thus, we used the ovarian cancer cell line SKOV3, in which NF‐kB signaling has been shown to be upregulated,^30^ to test the effect of INH14 on the cell‐migration ability (wound‐healing assay). The cells were grown overnight before a steady scratch was performed in each well. The cells were then incubated with vehicle; INH14; or 5Z‐7‐oxozeaenol as a control (TAK1 inhibitor^31^), which has been demonstrated to inhibit the migration potential. After 48 h, migration of the cells was observed by means of light microscopy. As shown in Figure 6 B, the wound closing of SKOV3 decreased for cells incubated with INH14, in comparison to those treated with the vehicle. However, the effect was lower than that observed with oxozeaenol at the same concentration.

NF‐kB signaling is constitutively activated in a variety of tumor cells and is associated with poor clinical outcome.^32^ Therefore, we wanted to investigate if INH14 could decrease this basal NF‐kB activation in SKOV3 cells. Cells seeded in 96‐well plates were transfected with *Elam.luc* and *Renilla* plasmids and, after overnight incubation with INH14, we measured NF‐kB activity. As observed in Figure 6 C, INH14 decreased the basal luciferase activity by 50 %. This result mirrored the results obtained by immunoblotting of SKOV3 cells incubated with INH14 (Figure 6 D). Treatment with the compound decreased IkBα degradation similarly to that with oxozeaenol.

In future studies, the effect of INH14 in combination with other chemotherapeutic agents, such as cisplatin, and its effect in other types of hematological and solid malignancies, in which the axis MyD88‐NF‐kB is constitutively activated,^33^ will be assessed.

### Modeling of INH14 in IKKβ

**Figure 7:**
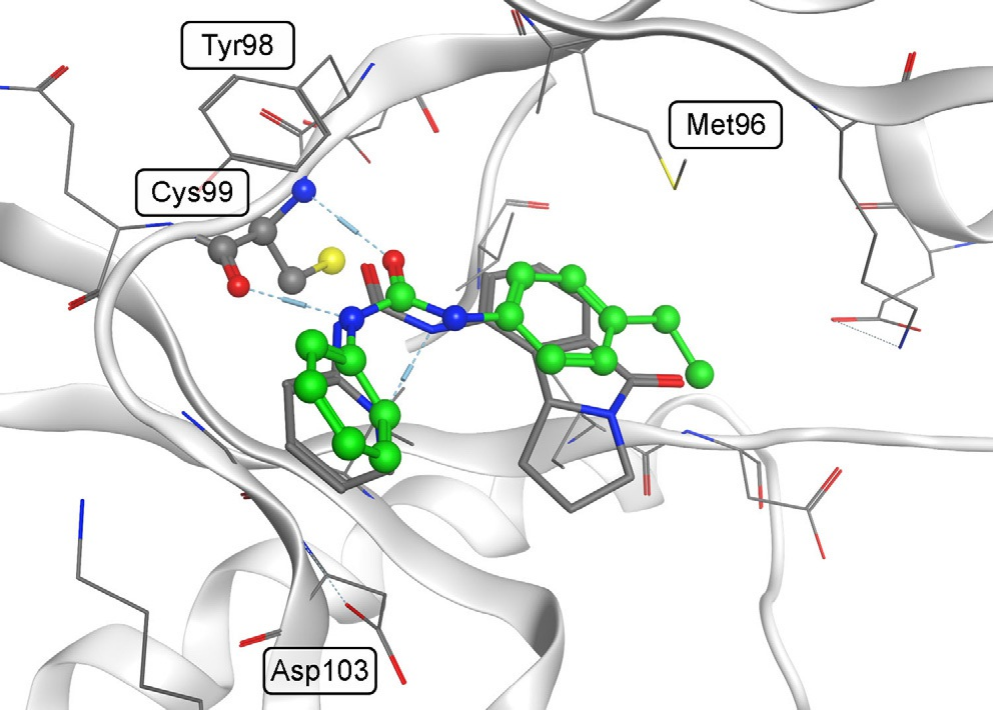
Predicted binding mode of INH14 (carbon atoms in green, oxygen atoms in red, and nitrogen atoms in blue) for IKKβ (protein structure originating from PDB ID: 4KIK; carbon atoms gray). Hydrogen bonds predicted to be formed with CYS99 are indicated as dashed lines with cylinders. The ligand shown with gray carbon atoms is the cyclin‐dependent kinase 4 (CDK4) inhibitor 1‐[(9bR)‐5‐oxo‐1,2,3,9b‐tetrahydrobenzo[f]pyrrolizin‐9‐yl]‐3‐pyridin‐2‐yl‐urea observed in an X‐ray structure with a CDK4 mimic CDK2 (PDB ID: 1GIH; aligned to 4KIK, protein structure not shown).

Docking studies on IKKβ strongly suggest that INH14 binds to the hinge region of the kinase (Figure 7), with the urea moiety forming two hydrogen bonds with the protein backbone (CYS99). The binding pose has strong similarity with that observed for an inhibitor of CDK2 (1‐[(9*bR*)‐5‐oxo‐1,2,3,9*b*‐tetrahydrobenzo[*f*]pyrrolizin‐9‐yl]‐3‐pyridin‐2‐yl‐urea, in complex with CDK2).^34^ Some uncertainty about the location of the ethyl group remains; the ligand might (also) bind in a flipped orientation.

## Conclusion

Our data indicate that the mechanism by which INH14 attenuates TLR2/TLR4/TNF‐R/IL‐1R signaling is through inhibition of IKKα/β. Docking studies strongly suggested the binding of the biaryl urea scaffold to the hinge region of the kinase. INH14 decreased in vivo TLR2‐induced inflammation, and in the future the inhibitory activity of INH14 in other animal models of inflammation will be studied. Our studies demonstrate that INH14 is a promising starting point for the development of anti‐inflammatory drugs.

## Experimental Section

**Cell culture**: HEK293 cells, RAW264.7 mouse macrophages, bone‐marrow‐derived macrophages, and the ovarian cancer cell line SKOV3 (ATCC collection and Sigma–Aldrich) were cultured in Dulbecco's modified Eagle's medium (DMEM; Sigma–Aldrich) with 10 % fetal bovine serum (FBS; Sigma–Aldrich) and 0.5 % (*v*/*v*) ciprofloxacin (Sigma–Aldrich) at 37 °C and 5 % CO_2_ atmosphere. The cell line HEK293‐TLR2 (kindly provided by D. T. Golenbock; Worcester, MA, USA) was cultured as described above, with the addition of puromycin (10 μg mL^−1^; Sigma–Aldrich). Human PBMCs were isolated from whole blood of healthy donors after volunteers′ informed consent. For the isolation of monocytes, blood was layered on Histopaque 1077 (Sigma–Aldrich) at a 1:1 ratio. The mix was centrifuged at 400 *g* for 30 min. Peripheral blood mononuclear cells (PBMCs) were collected and washed twice with phosphate‐buffered saline (PBS) and suspended in RPMI‐1640 media with 3 % FBS and 0.5 % ciprofloxacin (Sigma–Aldrich). Then, the cells were seeded in 96‐well plates at a density of 80×10^4^ cells per well and the next day the media was changed before treatment with different stimulants.

**Wound‐healing assay**: SKOV3 cells were seeded in 6‐well plate dishes at a density of 8×10^5^ cells per well. Then, the cell monolayer was scratched with a pipette tip (200 μL) to produce a narrow wound‐like slit. The growth medium was replaced with DMEM plus 2 % serum, and photographs were taken after 48 h with a 40× objective in a Leica DMI4000B microscope. The wound gap was measured with the ImageJ software. In each image, the wound spacing was measured in the upper, lower, and middle parts of the wound and the mean value was calculated. For each condition, four independent experiments were performed.

**Luciferase and ELISA assays**: Luciferase and ELISA (mTNF, hTNF; Biolegend, and R&D Systems) assays were performed as described previously.7a, 35

**Antibodies and reagents**: Anti‐IkB‐α, *p*‐p38, *p*‐JNK, pIKKα/β, Actin‐β, and horseradish peroxidase (HRP)‐conjugated anti‐mouse antibodies were obtained from Cell Signalling Technology; HRP‐conjugated anti‐rabbit was purchased from Sigma–Aldrich. Diacylated lipopeptide Pam2CSK4 (P2), triacylated lipopeptide Pam3CSK4 (P3), LPS, PolyI:C, TNFα, and IL‐1β were obtained from Invivogen. 5*Z*‐7‐Oxozeaenol was obtained from Sigma–Aldrich. INH14 was obtained from ChemBridge (ID 7140470; HPLC analysis in Figure S3). Stock solutions of the compounds were prepared in sterile DMSO (Sigma–Aldrich) at a concentration of 10 mm.

**Plasmids**: YFP‐MyD88, Flag‐IRAK1, Flag‐IKKα, Flag‐IKKβ, and Flag‐TRIF were purchased from Addgene. Additionally, *Elam.luc*, *kb3.luc*, *AP1.luc*., and *INFβ.luc* were kindly provided by D. T. Golenbock (Worcester, MA, USA); TAK1 and TAB1 expressing plasmids were kindly available from K. Matsumoto (Nagoya, Japan); pCMV‐Mal (PlasmidID) and *Renilla*‐pGL3 (Promega) were commercially available. Plasmids were prepared with the PureYield plasmid endotoxin‐free kit (Promega). The plasmid transient transfections were achieved with Fugene6 (Promega) by following the manufacturer's protocols.

**Kinase assays**: IKKα and IKKβ kinase assays (ADP‐Glo kinase assay) were purchased from Promega and used by following the manufacturer's instructions. Quantification of adenosine diphosphate (ADP) produced in the reactions (chemiluminescence) was measured with a Victor plate reader (PerkinElmer). The assay conditions are described in the legend of Figure 5.

**Immunoblotting**: HEK293‐TLR2 or SKOV3 cells (10^5^ cells per well) were grown in DMEM plus 10 % FBS overnight in 24‐well plates. The next day, the cells were treated as indicated. After stimulation, the cells were washed with PBS and then lysed with lysis buffer (150 mm NaCl, 0.1 % Tween, 20 mm Tris**⋅**HCl, pH 7.5) and a protease and phosphatase inhibitor mix (Roche Applied Science). The lysates were cleared by centrifugation at 10^4^ *g* for 10 min (4 °C). Equal amounts of the supernatant were separated by electrophoresis on SDS 10 % polyacrylamide gels and transferred to a polyvinylidene difluoride (PVDF) membrane (CarlRoth). The membrane was blocked for 1 h in 5 % nonfat milk in TBST (150 mm NaCl, 0.1 % Tween, 20 mm Tris**⋅**HCl pH 7.5). Then it was incubated overnight at 4 °C with the corresponding primary antibody and subsequently with HRP‐conjugated secondary antibody for 2 h. Immunoreactive proteins were detected by using Immobilon detection reagents (Millipore) and the Fusion analyzer imager (Vilber). Quantification was achieved with the FusionCapt software (Vilber).

**Cell viability**: Human primary monocytes (8×10^4^ cells per well) were seeded and incubated overnight with the compound, media control, or SDS (0.02 %). Then, the tetrazolium salt WST‐8 (2‐(2‐methoxy‐4‐nitrophenyl)‐3‐(4‐nitrophenyl)‐5‐(2,4‐disulfophenyl)‐2*H*‐tetrazolium monosodium salt; Sigma–Aldrich) was added, and the cells were incubated for an additional hour at 37 °C. During this period, the dehydrogenase activity of viable cells led to the production of the colored product (formazan). The cell viability was measured with a Victor plate reader (PerkinElmer) as an increase in the absorbance at *λ*=450 nm.

**Mice experiments**: The 8‐week‐old, male, pathogen‐free C57BL/6J mice (Charles River Laboratories) were maintained at the animal facility of the Medical University Innsbruck (12 h light/dark cycle; standard rodent chow and water available ad libitum). For lipopeptide‐induced inflammation, 5 μg g^−1^ of INH14 or vehicle was administered intraperitoneally. After 1 h, P2 (2.5 μg g^−1^) was injected, and tail vein blood (25 μL) was collected at that time point (0 h) and 2 h after. The blood was centrifuged at 5000 *g*, and the supernatant was frozen at −20 °C until further cytokine measurement by means of ELISA. Animal experiments were conducted according to national guidelines and European Community laws and were approved by the Committee for Animal Protection of the Austrian Ministry of Science.

**Statistical analysis**: GraphPad Prism (San Diego, CA, USA) was used to perform statistical analysis. Significance in the differences between data groups was assigned by using the Student t‐test.

**Docking studies**: Docking was performed with a structure of IKKβ originally bound with the staurosporine analogue K252a (PDB ID: 4KIK). The structure was prepared with the Structure Preparation wizard of Maestro.^36^ Following structure preprocessing, optimized hydrogen bonds were automatically assigned, any water molecules that formed less than three hydrogen bonds with non‐water molecules were removed, and the receptor structure was subjected to restrained minimization (default settings applied for all of these procedures were executed with the Structure Preparation wizard). The receptor grid for docking was generated for chain A, with the binding site defined by the location of the cocrystallized ligand (default settings). Docking was performed with Glide SP^37^ within Maestro (with default settings).

## Conflict of interest

*The authors declare no conflict of interest*.

## Supporting information

As a service to our authors and readers, this journal provides supporting information supplied by the authors. Such materials are peer reviewed and may be re‐organized for online delivery, but are not copy‐edited or typeset. Technical support issues arising from supporting information (other than missing files) should be addressed to the authors.

Supplementary
