# Supplementary material for: INH14, a Small‐Molecule Urea Derivative, Inhibits the IKKα/β‐Dependent TLR Inflammatory Response
Source: Chembiochem. 2019 Jan 29;20(5):710–7. doi: 10.1002/cbic.201800647 (PMC6680106; doi:10.1002/cbic.201800647)
Supplement: Supplementary file 1 — Supplementary [file CBIC-20-710-s001.pdf]

CHEMBIOCHEM

Supporting Information

INH14, a Small-Molecule Urea Derivative, Inhibits the IKKα/β-Dependent TLR Inflammatory Response

Meinrad Drexel+,[a] Johannes Kirchmair+,[b, c, d] and Sandra Santos-Sierra*[e]

cbic_201800647_sm_miscellaneous_information.pdf

SUPPORTING INFORMATION

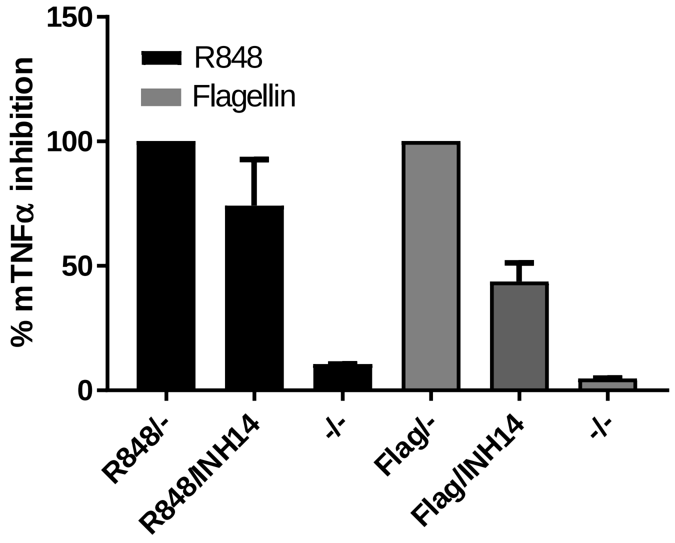

Supplementary 1: TNFα production by immortalized bone-marrow derived macrophages treated with INH4 (15 µM) and stimulated with the TLR7/8 ligand R848 (0.5 µg/ml) or the TLR5 ligand flagellin (1 µg/ml). After overnight incubation, the cytokine level was assessed by ELISA. The bars represent the mean and SEM of two independent experiments in triplicates.

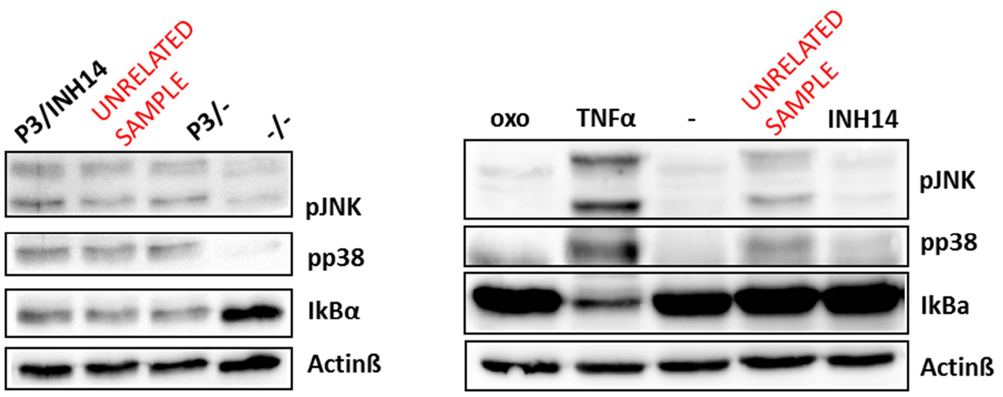

Supplentary 2: The immunoblot images appearing in Fig. 4B and Fig. 6D were cut out without further processing from the immunoblots here included. In the same experiments we tested several compounds in parallel, the lane labelled in red belongs to an unrelated compound and was cut-out.

CR00901923

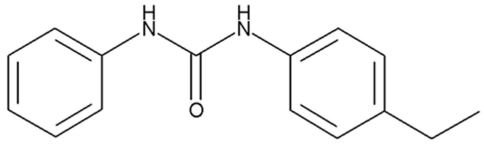

ID	7140470	240.3076	$C_{15}H_{16}N_2O$

```
Data File R:\HPLC\AUTO\CR009019\1GC-3401.D
Sample Name: CR009019P1-G-03
Instrument 1 12/10/2018 13:02:39
Column: Onyx C18 50x4.6mm | 3.75ml/min | Columns Reg Valve
Gradient: "A"->@2.0min->"B"(Hold 0.6min)->@0.2min->"A"->PostRun
PMP1, Solvent A          : 0.1%TFA, 2.5%AcN in H2O
PMP1, Solvent B          : 0.1%TFA in AcN
PMP1, Solvent C          : --NOT USED--
PMP1, Solvent D          : --NOT USED--
Ionization mode              :    API-ES Positive

Signal 1: ADC1 B, ELSD
Peak RetTime Type  Width     Area      Height     Area
  #   [min]        [min]   [mV*s]       [mV]        %
----|-------|----|-------|----------|----------|--------|
   1   1.662 MM    0.0454  737.82690  270.80154 100.0000
Totals :                   737.82690  270.80154


Signal 2: DAD1 A, Sig=300,200 Ref=off
Peak RetTime Type  Width     Area      Height     Area
  #   [min]        [min]   [mAU*s]     [mAU]        %
----|-------|----|-------|----------|----------|--------|
   1   1.602 MM    0.0791 5665.72803 1193.09253 100.0000
Totals :                  5665.72803 1193.09253


Signal 3: MSD1 TIC, MS File
Peak RetTime Type  Width     Area      Height     Area
  #   [min]        [min]                            %
----|-------|----|-------|----------|----------|--------|
   1   1.632 MM    0.0841 2.88475e7  5.71706e6  100.0000
Totals :                  2.88475e7  5.71706e6
```

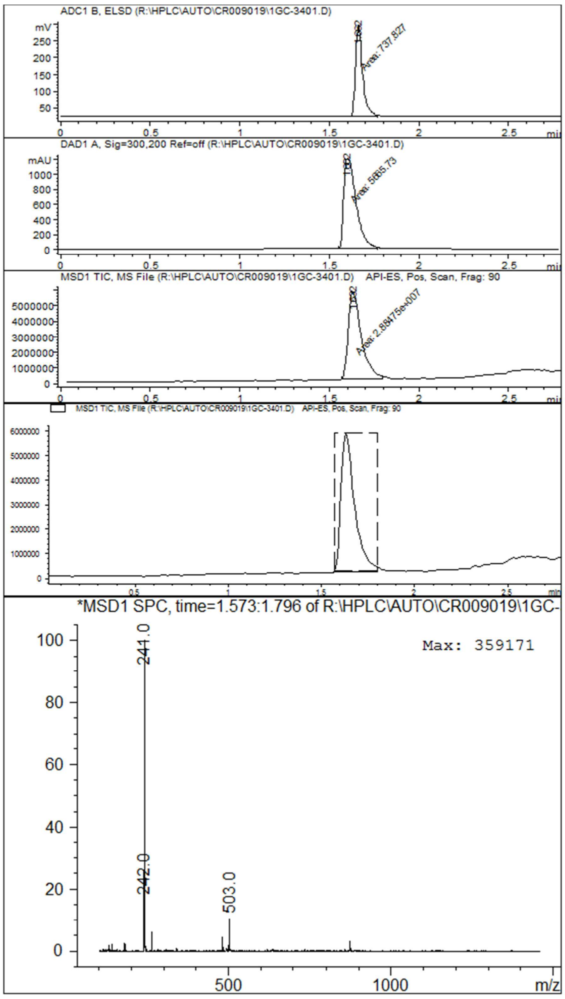

Supplentary 3: HPLC analysis of INH14 supplied by the vendor (Chembridge).
